# PPARγ deficiency results in reduced lung elastic recoil and abnormalities in airspace distribution

**DOI:** 10.1186/1465-9921-11-69

**Published:** 2010-06-02

**Authors:** Dawn M Simon, Larry W Tsai, Edward P Ingenito, Barry C Starcher, Thomas J Mariani

**Affiliations:** 1 Division of Respiratory Diseases, Children's Hospital Boston, Harvard Medical School, Boston, MA 02115, USA; 2 Department of Medicine, Brigham and Women's Hospital, Harvard Medical School, Boston, MA 02115, USA; 3 Department of Biochemistry, University of Texas Health Center, Tyler, TX 75708, USA; 4 Division of Neonatology and Center for Pediatric Biomedical Research, Department of Pediatrics, University of Rochester Medical Center, Rochester, NY, USA; 5 Division of Pulmonary, Allergy, Cystic Fibrosis and Sleep, Emory University School of Medicine, 2015 Uppergate Dr. Suite 338, Atlanta, GA 30322, USA

## Abstract

**Background:**

Peroxisome proliferator-activated receptor (PPAR)-γ is a nuclear hormone receptor that regulates gene expression, cell proliferation and differentiation. We previously described airway epithelial cell PPARγ deficient mice that develop airspace enlargement with decreased tissue resistance and increased lung volumes. We sought to understand the impact of airspace enlargement in conditionally targeted mice upon the physio-mechanical properties of the lung.

**Methods:**

We measured elastic recoil and its determinants, including tissue structure and surface forces. We measured alveolar number using radial alveolar counts, and airspace sizes and their distribution using computer-assisted morphometry.

**Results:**

Air vs. saline-filled pressure volume profiles demonstrated loss of lung elastic recoil in targeted mice that was contributed by both tissue components and surface tension, but was proportional to lung volume. There were no significant differences in surfactant quantity/function nor in elastin and collagen content between targeted animals and littermate controls. Importantly, radial alveolar counts were significantly reduced in the targeted animals and at 8 weeks of age there were 18% fewer alveoli with 32% more alveolar ducts. Additionally, the alveolar ducts were 19% larger in the targeted animals.

**Conclusions:**

Our data suggest that the functional abnormalities, including loss of recoil are secondary to altered force transmission due to differences in the structure of alveolar ducts, rather than changes in surfactant function or elastin or collagen content. These data further define the nature of abnormal lung maturation in the absence of airway epithelial cell PPARγ and identify a putative genetic determinant of dysanapsis, which may serve as a precursor to chronic lung disease.

## Background

Genetic heritability of airway structure and function in humans has been described [1,2]. Further, variation in lung structure [3] and function [4-6] also exists in genetically distinct mouse strains. Green et al coined the term "dysanapsis" to describe inter-individual discrepancy between parenchyma and airway size [7]. They postulated that these differences have an embryological basis that reflects physiologically normal, though disproportionate, airway and parenchyma growth. Martin et al determined that dysanapsis did indeed exist in early childhood and remained uniform throughout growth [8]. These variations in airway-parenchymal relationships may influence the development of obstructive airway disease [7,9]. As heritability for airway function exists [1], it is likely that genetic factors contribute to dysanaptic lung growth resulting in a distribution of normal lung function. However, we currently lack a complete understanding of the normal distribution in lung function that arises from variation in lung growth and the genes responsible.

Peroxisome proliferator-activated receptor (PPAR)-γ is a nuclear hormone receptor that regulates gene expression, cell proliferation and differentiation. PPARγ is one of three PPARs, which cumulatively function as regulators of cellular lipid trafficking and metabolism [10]. They function at the transcriptional level, whereby upon activation by an appropriate ligand, PPARs form an obligate heterodimer with RXRs (cis-retinoic acid receptors) to recruit nuclear receptor coactivators to specific promoter elements, termed peroxisome proliferator response elements, and modulate gene transcription [10]. PPARγ appears to have multiple functions, including regulating organ development [11,12], cellular differentiation [13-15], organ inflammation [16-18] and cellular survival [19,20]. PPARγ function is essential for survival and its deficiency is associated with death at mid-gestation, prior to lung formation, with severe cardiovascular anomalies arising from insufficient differentiation of placental cytotrophoblasts [11]. Within the lung, PPARγ expression has been reported in the epithelium [17,20], smooth muscle [20,21], endothelium [22], macrophages [19], eosinophils [16] and dendritic cells [23].

We previously described mice with conditional PPARγ deficiency within the conducting airway epithelium [24]. These conditionally targeted mice develop airspace enlargement concurrent with alveolarization, indicating abnormal or insufficient postnatal lung maturation. The observed pathology is not progressive with ageing. Airspace enlargement is accompanied by alterations in lung physiology, including increased lung volumes and decreased tissue resistance. Our data suggest that this abnormality may be secondary to alterations in epithelial cell differentiation, which leads to disruption of the normal epithelial-mesenchymal interactions necessary for establishing parenchymal structure and function. PPARγ is a major regulator of cellular lipid homeostasis, and has previously been shown to influence surfactant protein gene expression [25]. Therefore, alterations in lung structure and function in conditionally targeted mice could alternatively be secondary to changes in airspace surface tension contributed by surfactant.

We sought to better understand the impact of the airspace enlargement in conditionally targeted mice upon the physio-mechanical properties of the lung, such as elastic recoil, and how surface tension and tissue elasticity contribute to total lung recoil. Recoil is the tendency of a tissue to return to its resting shape when an external force is removed [26]. It is a function of various components of tissue structure and surface forces [27].

Here, we report that alterations in lung structure and physiology in airway epithelial cell PPARγ targeted mice are accompanied by a loss of absolute tissue elastic recoil. These recoil effects are not attributable to changes in surfactant quantity or function, or extracellular matrix content or distribution. However, we find a significant abnormality in the distribution of airspace size, with mature conditionally targeted animals displaying a reduction in alveolar number and increase in alveolar duct number and size.

## Methods

### Animals

The generation and use of genetically modified animals was performed according to approved Harvard Medical School Institutional Animal Care and Use Committee protocols. Airway epithelium-specific PPARγ deficient mice were generated as described previously [24]. Briefly, airway epithelium-specific PPARγ deficient mice were generated by breeding transgenic mice expressing Cre recombinase driven by a CC10 promoter (CCtCre mice) with mice harboring loxP sites flanking exon 2 of the PPARγ gene (PPARγ floxed mice) as described previously. We generated conditionally targeted animals (PPARγ^floxed/floxed^, Cre^+^) and littermate controls (PPARγ^floxed/floxed^, Cre^-^) by appropriate matings. Animals were genotyped for the presence of the Cre and PPARγ floxed alleles by tail biopsy, which provides a presumptive genotype predicting conditional targeting and confirmed by postmortem analysis of lung DNA. Targeted animals were specifically defined as those displaying the recombined allele, identified at 400 bp. All studies were performed on 8-12 week old mice unless otherwise noted comparing conditionally targeted animals with littermate controls.

### Physiology

The contributions of surface tension (P_γ_) and tissue elasticity (P_tis_) to lung recoil (P_el_) were determined by measuring recoil with deflation pressure-volume curves in degassed lungs filled first with air and then with saline as previously described [24,27]. Recoil pressure in the saline-filled state is equal to the tissue contribution to recoil (P_tis_). Recoil pressure in the air-filled state (P_el_) is equal to the combined contributions from tissue (P_tis_) and surface tension (P_γ_). P_γ_, which includes surface tension contributions from both the alveoli and alveolar ducts, is calculated from the expression P_el _= P_tis _+ P_γ _at iso-volume [28].

### Elastin histochemistry

The left lung was inflated and fixed at a constant pressure with 10% buffered formalin then embedded parasagitally in paraffin as previously described [24]. A modified Hart's stain was used to identify elastin fibers by incubating the slides in working solution overnight and counterstained with tartrazine as described previously [29]. Briefly, sections of paraffin-embedded, formalin-fixed tissue were deparaffinized through xylene and hydrated through graded washes of ethanol. They were incubated in working solution overnight: 10 ml Resorcin-fuscin stock, 100 ml 70% ethanol and 2 ml hydrochloric acid. After several washes in water, slides were counterstained with tartrazine (0.5 gm tartrazine, 200 ml water and 0.5 ml acetic acid) for 3 minutes, rinsed in water, dehydrated and placed in xylene prior to coverslipping.

### Elastin and collagen biochemistry

Whole lung tissue obtained from the right lung was flash frozen in liquid nitrogen and thawed immediately prior to analysis. Following acid hydrolysis, the samples were assayed for the unique elastin fiber cross-link desmosine (Des) by radioimmunoassay and the collagen marker hydroxyproline (HP) by amino acid analysis, as previously described [30]. Total protein in the hydrolysates was determined as described previously [31]. Desmosine and hydroxyproline content were normalized for total protein content (P) of the samples.

### Surfactant analysis

A subset of animals underwent bronchoalveolar lavage (BAL) with 1 ml of saline × 3. The lung lavage fluid was pooled for each animal, and cells removed by low speed (250 × g for 6 minutes) centrifugation. The surfactant pellet was isolated by high speed centrifugation (14,000 × g for 20 minutes), and total phospholipid content determined as previously described [32]. Values are representative of whole lung and are normalized for unit volume. Samples were brought up to 1 mg/ml final phospholipid concentration in 5 mM CaCl_2_. Surface film interfacial properties were recorded using a pulsating bubble surfactometer (Electronetics Corp, Tampa, FL) at 20 oscillations/min, 37°C, and 100% relative humidity as previously described [32]. Films were characterized in terms of minimum, maximum, and equilibrium surface tensions recorded at steady state.

### Lung morphometry

We collected data describing the sizes of individual airspaces (airspace area) using computerized morphometry methods as previously described [24,29]. Briefly, sections of inflated formalin-fixed lung tissue embedded parasagitally were stained with a modified Gill's hematoxylin for 24 hours. Ten random high-powered fields from one section for each animal were captured using MetaMorph 4.6.5 software (Molecular Devices Corporation, Sunnyvale, CA) and analyzed with Scion imaging (Scion Corporation, Frederick, MD) to calculate the airspace area. The mean size and frequency of airspaces per high powered field (hpf) were calculated by binning the airspace areas based on the expected size of alveoli (250-2,000 μm^2^) and alveolar ducts (≤ 5,000 μm^2^), which were determined based upon empirical values of airspace sizes in control animals (See Additional File 1).

Hematoxylin and eosin stained slides of inflated, fixed lung tissue were analyzed to calculate radial alveolar counts (RAC) using the method originally described by Emery and Mithal [33]. Briefly, the number of alveoli transected by a line drawn perpendicular to the pleural surface from a terminal respiratory bronchiole to the nearest pleura was counted. RAC were performed for all terminal respiratory bronchioles present in one section for each animal and the mean RAC per terminal bronchiole calculated.

### Statistical analysis

Two-tailed unpaired Student's t-test was performed for individual comparisons between conditionally targeted and littermate control animals.

## Results

### Airway epithelial cell PPARγ targeted mice display reduced iso-volume lung elastic recoil

The contribution of surface tension and tissue forces to lung recoil can be assessed by measuring deflation pressure-volume curves of the degassed lung filled first with air, followed by saline [27,28]. Air-filled P-V data measures the combined effects of surface tension (P_γ_)and tissue elasticity (P_tis_) to recoil (P_el_). Saline-filled data measures the tissue contribution (P_tis_) to lung elasticity. The difference between these is the surface film contribution (P_γ_).

**Figure 1:**
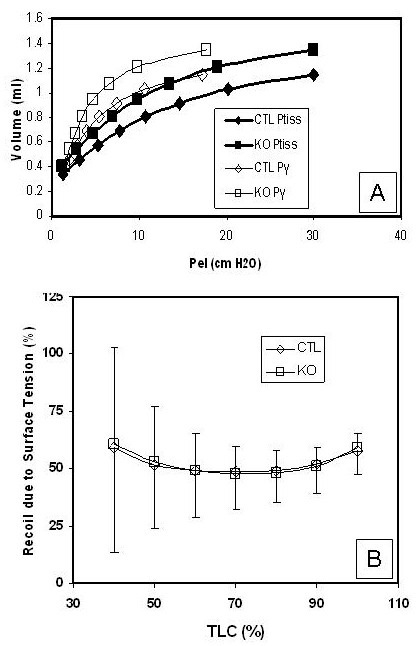
**Lung elastic recoil**. The relative and absolute contributions of surface tension (P_γ_) and tissue recoil (P_tis_) to lung elasticity (P_el_) in conditionally targeted (KO; n = 8) (red) and littermate control (CTL; n = 8) (blue) mice were evaluated from air- and saline-filled pressure-vs-volume profiles. **A**, Absolute lung elasticity (P_el_) measured as tissue recoil (P_tis_) and surface tension (P_γ_) are reduced in conditionally targeted mice. **B**, Proportion of lung elasticity resulting from surface tension corrected for lung volume is similar between conditionally targeted and littermate control mice. Error bars represent standard deviation.

The relative and absolute contributions of surface tension and tissue recoil to lung elasticity in conditionally targeted (KO; n = 8) and littermate control (CTL; n = 8) mice were evaluated using this approach. Quasi-static deflation pressure-volume (QSDPV) data were fit to the exponential expression of Salazaar-Knowles, V(P) = V_max _- (V_max _- V_min_) e^-kP^, where V_max _is defined as the theoretical lung volume predicted for infinite distending pressure, V_min _the lung volume at 0 distending pressure, k the parameter that determines the curvature of the exponential function describing the relationship between pressure and volume, and P the transpulmonary pressure. Results for each group were summarized in terms of mean values for V_max_, V_min_, and k and representative group mean P-V relationships constructed. Figure 1A depicts absolute lung volume as a function of distending pressure for the air-filled state (P_el_) in control (blue) and conditionally targeted animals (red). At iso-volume, lung recoil pressure is significantly reduced in KO mice relative to controls, as demonstrated by a leftward shift in the QSDPV curve. Results further indicate that the iso-volume reductions in overall lung recoil in the KO group are the result of decreases in both tissue (P_tis_) and surface tension (P_γ_) forces relative to control animals, as both curves show a relative leftward shift. Normalized to lung volume, the relative contributions of surface (Figure 1B) and tissue (data not shown) forces to overall recoil in the conditionally targeted mice are similar to controls.

### Airway epithelial cell PPARγ targeted mice have normal collagen and elastin fiber content

**Figure 2:**
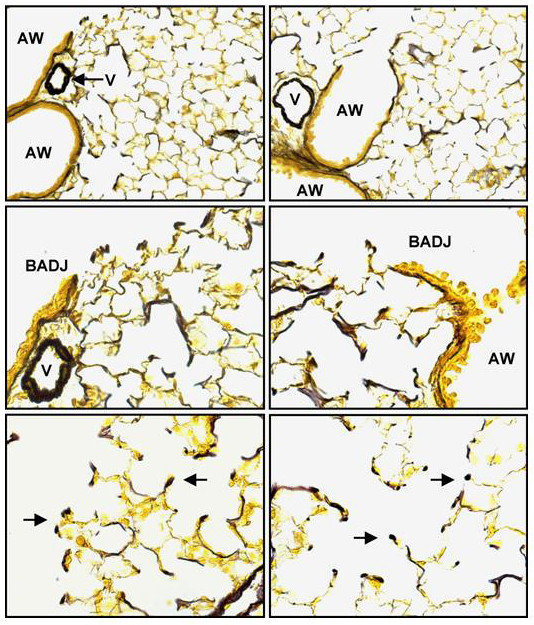
**Distribution of lung elastic fibers**. Hart's elastin stain was performed on inflated fixed lung tissue from 8 week old conditionally targeted (**B, D, F**) and littermate control (**A, C, E**) mice. Low magnification images (**A, B**) reveal elastin deposition most prominently in vascular (V) media and conducting airway (AW) walls. High magnification images (**C-F**) reveal elastin distribution at the terminus of the conducting airway wall (the bronchiole-alveolar duct junction; BADJ) and in the parenchyma, with fibers concentrated at the tips of alveolar septae (arrow).

The observed airspace enlargement and physiological changes in the lungs of conditionally targeted mice could be explained by defects in airway-airspace tethering, primarily contributed by the extracellular matrix (ECM). Elastin histochemistry did not show any apparent changes in quantity, distribution or orientation of elastin fibers in the lungs of conditionally targeted or littermate controls at 8 weeks of age (Figure 2). Similar to controls, the elastin fibers of conditionally targeted animals were most abundant in the vascular intima and, to a lesser extent, in the sub-epithelial layer of conducting airways (Figure 2A-D). Conditionally targeted mice also showed a normal distribution of elastin fibers in the parenchyma, with fibers concentrated at the tips of alveolar septae (Figure 2E, F).

Biochemical analysis for desmosine (Des) showed no difference in elastin content when corrected for total protein content (P) in conditionally targeted animals compared with littermate controls (534.1 ± 81.5 vs. 516.6 ± 62.5 pmDes/mgP, respectively). Similarly, biochemical analysis for hydroxyproline (HP) showed similar collagen content between conditionally targeted and control animals (30.5 ± 4.2 vs. 30.3 ± 4.3 nmHP/mgP, respectively).

### Normal surfactant quantity and function in airway epithelial cell PPARγ targeted mice

As the content and distribution of ECM appeared not to be the cause of altered lung mechanics in conditionally targeted mice, we investigated surfactant quantity and function. Surfactant was isolated from the lungs of mature conditionally targeted (n = 9) and littermate control (n = 7) mice by bronchoalveolar lavage. Analysis of lavage surfactant demonstrated a similar content of total lung phospholipids (228 ± 48.2 vs. 214 ± 14.7 ug, respectively).

Surfactant function was characterized in terms of interfacial properties measured by pulsating bubble surfactometry. Characterization was expressed in terms of maximum (γ_max_), minimum (γ_min_), and equilibrium (γ_equil_) surface tensions measured under dynamic and static conditions. There were no differences in γ_max _(37.67 ± 2.7 vs. 37.14 ± 1.8 dyn/cm), γ_min _(9.44 ± 4.7 vs. 10.29 ± 3.5 dyn/cm) or γ_equil _(25.56 ± 0.7 vs. 25.57 ± 0.8 dyn/cm) in conditionally targeted animals compared with littermate controls.

### Abnormal distribution of airspace size in airway epithelial cell PPARγ targeted mice

**Figure 3:**
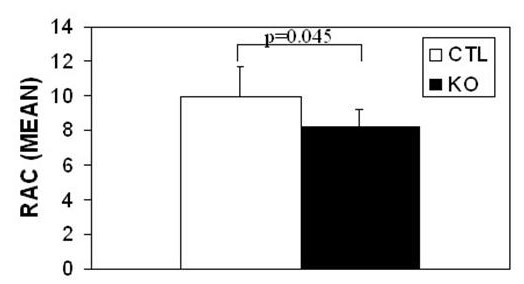
**Radial alveolar counts**. Radial alveolar counts (RAC) were performed on hematoxylin and eosin-stained tissue sections from conditionally targeted (n = 7) and littermate control (n = 8) mice at 8 weeks of age. There is a significant 17% reduction in the number of alveoli in conditionally targeted mice (mean number of 8.2 vs. 9.9, p = 0.045). Error bars represent standard deviation.

Given the absence of significant changes in either ECM structure or surfactant quantity/function, we further characterized airspace morphometry. We previously described airspace enlargement in conditionally targeted mice, with increases in both mean chord length (distance between alveolar walls) and airspace area (size of individual airspaces) [24]. We observed the appearance of numerous alveoli of normal size in conditionally targeted lungs, with an apparent reduction in alveolar number (per high power field). To directly test for changes in alveolar number, we performed radial alveolar counts (RAC). As presented in Figure 3, these data confirmed that the number of alveoli was reduced by 17% in conditionally targeted animals (mean number of 8.2 RAC/terminal bronchiole vs. 9.9 RAC/terminal bronchiole, p = 0.045).

**Figure 4:**
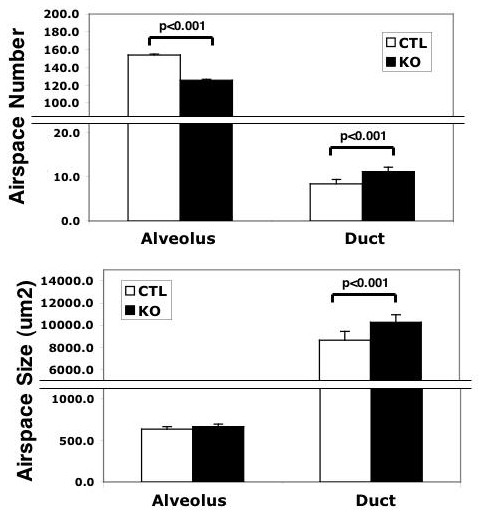
**Analysis of airspace number and size**. Computerized morphometry was used to determine the sizes of individual airspaces in 8 week old conditionally targeted (n = 12) and littermate control (n = 11) mice. **A**, Frequency of individual airspaces corresponding to the size of alveoli or alveolar ducts. **B**, Mean size of airspaces defined as alveoli or alveolar ducts. Consistent with RAC, at 8 weeks of age there are 18% fewer alveoli with 32% more alveolar ducts in conditionally targeted animals. Additionally, alveolar ducts are 19% larger, while alveoli are similar in size (4% larger) in targeted animals compared with littermate controls. Error bars represent standard deviation.

In order to further define the nature of these changes, we analyzed the distribution of airspaces of varying size (See Additional File 2). Conditionally targeted lungs displayed a consistent decrease in the frequency of smaller airspaces (<1000 μm^2^), and a consistent increase in the frequency of airspaces >1000 μm^2 ^(See Additional File 2). We categorically defined alveoli as airspaces of 250-2,000 μm^2^, and alveolar ducts as airspaces of >5,000 μm^2 ^(See Additional File 1 for explanation). We observed an 18% decrease in relative alveolar number per high power field (mean number of 126/hpf vs. 154/hpf, p < 0.0001) (Figure 4A) with a minimal change (4% increase) in mean alveolar size (665 μm^2 ^vs. 638 μm^2^, p = 0.047) (Figure 4B) in the conditionally targeted mice. This is in good agreement with results from RAC. Of further interest, we found a 32% increase in the relative number of alveolar ducts per high power field (mean number of 11.1/hpf vs. 8.4/hpf, p < 0.0001) in the conditionally targeted animals compared with littermate controls (Figure 4A). Additionally, alveolar ducts were 19% larger (10,274 μm^2 ^vs. 8,658 μm^2^, p < 0.0001) in the targeted animals (Figure 4B).

### Ontogeny of airspace distribution in airway epithelial cell PPARγ targeted mice

**Figure 5:**
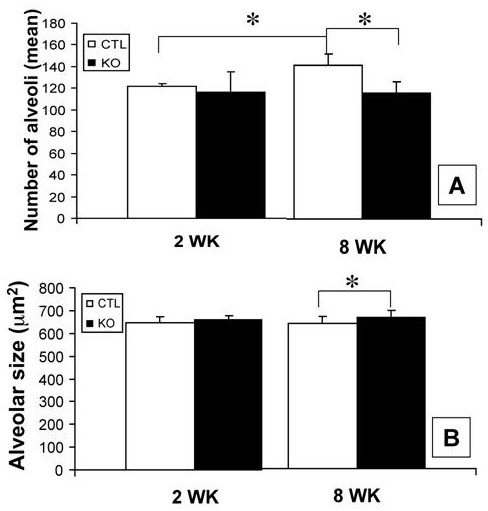
**Ontogeny of airspace distribution**. Computerized morphometry was used to determine the ontogeny of airspace size and distribution across postnatal maturation in conditionally targeted and littermate control mice. **A**, Alveolar frequency at 2 and 8 weeks of age. **B**, Alveolar size at 2 and 8 weeks of age. **C**, Alveolar duct frequency at 2 and 8 weeks of age. **D**, Alveolar duct size at 2 and 8 weeks of age. There was a significant (27%) increase in the number of alveoli in the control animals that was not seen in the targeted animals between 2 and 8 weeks of age. There was a significant (15%) increase in the number of alveolar ducts in the targeted animals that was not seen in the control animals between 2 and 8 weeks of age. There was a significant (11%) increase in the size of the alveolar ducts in the targeted animals that was not seen in the control animals between 2 and 8 weeks of age. * p-value is less than 0.05. Error bars represent standard deviation.

As we previously reported that airspace enlargement in conditionally targeted mice occurs postnatally, we assessed the ontogeny of airspace distribution (Figure 5). At 2 weeks of age, there was no difference in the frequency or size of alveoli and alveolar ducts between conditionally targeted and littermate control animals (Figure 5A-D) consistent with our previous report (15). When assessing changes in airspaces from 2 to 8 weeks of age, there was no significant change in the mean size of alveoli for either the conditionally targeted animals or their littermate controls (Figure 5B) though there is a 27% increase in the relative frequency of alveoli per high power field in the controls (mean number of 121/hpf vs. 154/hpf, p < 0.0001) (Figure 5A). While this increase in the number of alveoli is not seen in the conditionally targeted animals (116/hpf vs. 126/hpf, p = 0.197), there is a 14% increase in the relative number of alveolar ducts per high power field (mean number of 9.69/hpf vs. 11.1/hpf, p = 0.029) (Figure 5C) as well as an 11% increase in the size of alveolar ducts during this time (mean size of 9,289 vs. 10,274 μm^2^, p = 0.01) (Figure 5D).

## Discussion

We sought to understand the role of PPARγ in the regulation of lung development and homeostasis in gene targeted mice. Due to the early fetal lethality of complete PPARγ deficiency [11], we used a conditional targeting strategy where we deleted PPARγ specifically within the conducting airway epithelial cells using a novel line of mice expressing a non-regulatable form of Cre recombinase driven by the rat CC10 promoter [24]. The resultant conditionally targeted animals display abnormal lung maturation with the development of enlarged airspaces compared with littermate controls. Tissue resistance was significantly lower in conditionally targeted mice, consistent with differences in parenchymal geometry, differences in surface film properties, and/or differences in the constitutive mechanical properties of structural tissue elements. The current line of investigation was undertaken to better characterize the physiologic consequences of airspace enlargement due to epithelial cell PPARγ deficiency and to define the underlying basis of these abnormalities.

There have been reports suggesting that Cre recombinase is toxic to mammalian cells [34]. Considering our model utilizes Cre recombinase to cause cell specific gene deletion, we controlled for this as a confounding factor by evaluating airspace size in mice containing Cre recombinase. It is important to note that heterozygous mice (PPARγ^floxed/WT^, Cre^+^) and the CCtCre transgenic mice were analyzed and found to have comparable airspace size with the conditionally targeted littermate controls [24].

Recoil is the tendency of a tissue to return to its resting shape when an external force is removed [26] and is determined by the combined effects of tissue and surface tension recoil, with tissue contributing approximately 1/3 of recoil and surface tension 2/3 of recoil over the vital capacity range. The geometry and mechanical properties of lung structure, in particular alveolar ducts, contribute to the tissue effects on recoil [35]. Additionally, the lungs contain a network of elastin and collagen fibers, which resist expansion and contribute to the elastic recoil properties. Recoil is also affected by surface forces at the air-liquid interface. Surfactant lowers this surface tension to reduce the work of lung expansion with inspiration and prevent complete alveolar collapse at end-expiration. We characterized the absolute and relative contributions of each of these determinants of recoil in conditionally targeted mice, to assess if observed differences in lung morphometry represent developmental dysanapsis due to extended lung growth, with relatively preserved lung physiology when normalized to total lung capacity.

We observed that conditionally targeted mice have enlarged lungs that display reduced recoil properties, but were proportional when normalized to total lung volume. Such changes could be explained by abnormalities in lung ECM-mediated tethering. We previously reported changes in the basal expression levels of specific collagen (Col1, Col3) and elastin genes in adult conditionally targeted lung tissue, after the time when most of the matrix has been produced and has accumulated. However, analysis of lung elastin and collagen protein content indicated there were no significant differences in the distribution of ECM in the lungs of conditionally targeted mice. These biochemical methods measure the accumulation of protein throughout the heterogeneous tissue over time, and are a more robust indication of the physiological contributions of the ECM. The data reported in the current study thus clarifies that differences in expression of these genes at this time-point are not sufficient to alter whole lung tissue content of these markers. Thus, changes in lung recoil could not be accounted for by a decrease in total tissue content of the two major constituents of the lung's ECM. The ECM of the lung is highly complex, consisting of dozens of collagen molecules and numerous molecules necessary for elastin fiber formation, with unique distributions. We do not report, nor have we tested, for changes in the local distributions (e.g., vessels vs. alveoli or basement membrane vs. interstitium) of specific molecules. Whether our previously defined changes in expression reflect ongoing repair, alterations in homeostatic mechanisms or affect local distributions of ECM components is currently unknown.

Since PPARγ contributes to cellular lipid metabolism and trafficking, it is also reasonable to hypothesize that a deficiency in PPARγ may affect surfactant lipid production and/or secretion. Analysis of surfactant isolated from the lungs of conditionally targeted mice did not reveal any differences in absolute phospholipid quantity or surfactant surface tension. These data suggest that abnormalities in surface forces from surfactant are not a direct cause for the loss of elastic recoil in the conditionally targeted animals and do not support a role for airway epithelial cell PPARγ in regulating surfactant expression *in vivo*.

It is appreciated that abnormalities in alveolar duct structure, distribution or function can alter surface forces and ultimately affect elastic recoil [27]. Computerized quantitative morphometric analysis revealed an abnormality in the distribution of airspaces in the conditionally targeted mice representative of alveoli and alveolar ducts. We identified a greater than 30% relative increase in alveolar duct number and nearly 20% relative increase in mean alveolar duct size. The accepted technique of radial alveolar counts (RAC) confirmed an almost 20% decrease in the relative number of alveoli. While changes in the sizes of individual airspaces in conditionally targeted lungs is likely affected by reduced tissue resistance, this would only explain our observations if individual alveoli in conditionally targeted lungs were increased at least 2-fold in size at the same pressure (25 cm H20). We conclude that these alterations in airspace distribution lead to iso-volume loss of elastic recoil and an overall increase in lung volumes in conditionally targeted animals.

Differences in lung structure and/or function may be a contributing factor for the development of lung disease [36]. Airway-parenchymal relationships critically influence the development of obstructive airway disease [7,9]. Litonjua et al [37] and Parker et al [38] determined that the FEF25-75/FVC ratio, a measure of dysanapsis, was negatively associated with the degree of methacholine airway responsiveness, implying small airway size increases the risk of airway obstructive disease. Airway hyperresponsiveness is related to the development of asthma and may be genetically determined [39,40]. Additionally, the prevalence of asthma and wheezing negatively correlates with airway size as evidenced by increased prevalence in prepubertal males [41] who have smaller airways [9,42] compared with females. Silverman et al reported that genetic determinants of lung function likely represent determinants of COPD and asthma [43]. While the phenotype of our conditionally targeted mice is more representative of normal variation, preliminary studies suggest they are more susceptible to the development of experimental lung disease [44].

## Conclusions

We report airway epithelial cell PPARγ deficiency results in airspace enlargement accompanied by a reduction in total lung elastic recoil. Our data indicate that these physiological abnormalities are secondary to altered force transmission due to differences in the structure of the lung parenchyma, rather than changes in elastin or collagen content or surfactant function. These data further clarify the special role of lung epithelial cell PPARγ in contributing to the establishment of normal airspace structure. Finally, our study describes how genetics can contribute to the determination of lung structure and function, which may predispose to chronic lung disease.

## Competing interests

The authors declare that they have no competing interests.

## Authors' contributions

DMS organized the studies, performed or assisted with all experiments and contributed to writing the manuscript. LWT performed physiology and lipid biochemistry experiments. EPI conceived the studies and participated in physiology and lipid biochemistry experiments and contributed to data interpretation. BCS performed extracellular matrix biochemistry experiments. TJM conceived the studies, contributed to data analysis and interpretation, and wrote the manuscript. All authors read and approved the manuscript.

## Supplementary Material

Additional file 1 **Supplemental Methods**. This file provides detailed methodology on the computer-assisted determination of alveolus and alveolar duct number and size. Click here for file

Additional file 2 **Supplemental Figure**. This figure shows the frequency distribution for airspaces of individual sizes for both control and conditionally targeted mice. Click here for file
