# Supplementary material for: PPARγ deficiency results in reduced lung elastic recoil and abnormalities in airspace distribution
Source: Respir Res. 2010 Jun 2;11(1):69. doi: 10.1186/1465-9921-11-69 (PMC2889874; doi:10.1186/1465-9921-11-69)
Supplement: Additional file 1 — Supplemental Methods. This file provides detailed methodology on the computer-assisted determination of alveolus and alveolar duct number and size. [file 1465-9921-11-69-S1.DOC]

***Computer-assisted determination of alveolus/duct number and size.***

We calculated the area of individual airspaces from 10 random high-powered fields of each animal using Scion analysis of MetaMorph images as described in Methods. Each field generated a distribution of airspace sizes that was a representation of the overall lung structure in each animal (Additional File 2). We excluded airspaces bordering the edge of the micrographs, as it was not possible to define their true size. Typically, each field contained greater than 200 individual airspaces, ranging in size from less than 25 μm^2^ to greater than 10,000 μm^2^. These quantitative data provide an imperfect representation of actual airspace sizes, due to the level and angle at which the section bisected the airspace. Therefore, we transformed these data into categorical values representative of airspaces for frank alveoli or alveolar ducts. We objectively determined the range of airspace sizes representative of true alveoli, and those representing alveolar ducts, by visual examination of the sizes of histological structures associated with individual airspaces in control lung samples. Airspace measurements less that 250 μm^2^ were often from structures that did not represent recognizable airspaces, more reflective of artifacts or small vessel lumen. Structures greater than 2000 μm^2^ typically appeared to be composed of two or more adjoining alveoli. However, airspaces between 2000 and 5000 μm^2^ were often of complex structure and difficult to categorize as true alveolar ducts with high confidence. We used these observations to imposed categorical definitions to divide airspace area measurements into unique, non-overlapping ranges representative of alveoli (250-2,000 μm^2^) and alveolar ducts (>5,000 μm^2^). From the categorical data, we were able to determine both the number and size of alveoli and alveolar ducts in each field.
