# Supplementary material for: PPARγ deficiency results in reduced lung elastic recoil and abnormalities in airspace distribution
Source: Respir Res. 2010 Jun 2;11(1):69. doi: 10.1186/1465-9921-11-69 (PMC2889874; doi:10.1186/1465-9921-11-69)

**Supplemental Figure 1. Distribution of airspace area measurements.** The size of individual airspaces in (μm^2^) was determined in high-powered micrographs of Gills-stained lung sections as described in Methods. Shown is the frequency distribution for airspaces of individual sizes for both control and conditionally targeted (KO) mice. Bins are composed of measurements greater than the previous bin up to the value listed (e.g., bin “1000” represents measurements from 801-1000 μm^2^). A change in the distribution of airspace sizes between genotypes can be readily observed, with controls having more airspaces <1000 μm^2^ and conditionally targeted mice having more airspaces >1200 μm^2^.


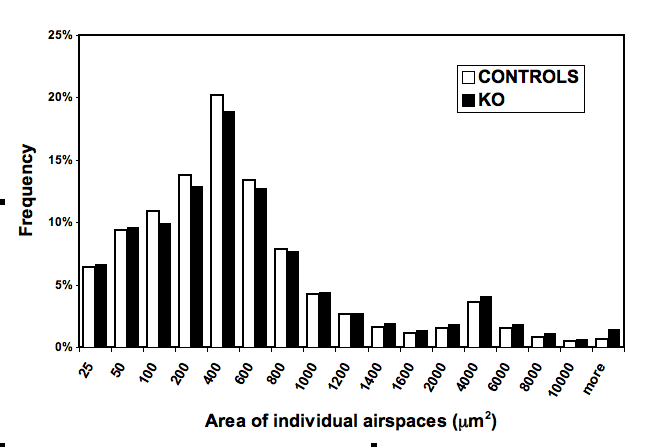

Supplement: Additional file 2 — Supplemental Figure. This figure shows the frequency distribution for airspaces of individual sizes for both control and conditionally targeted mice. [file 1465-9921-11-69-S2.DOC]
